# Semiquantitative Evaluation of Extrasynovial Soft Tissue Inflammation in the Shoulders of Patients with Polymyalgia Rheumatica and Elderly-Onset Rheumatoid Arthritis by Power Doppler Ultrasound

**DOI:** 10.1155/2017/4272560

**Published:** 2017-02-15

**Authors:** Takeshi Suzuki, Ryochi Yoshida, Akiko Okamoto, Yu Seri

**Affiliations:** ^1^Division of Allergy and Rheumatology, Japanese Red Cross Medical Center, Tokyo, Japan; ^2^Division of Rheumatology, Mitsui Memorial Hospital, Tokyo, Japan

## Abstract

*Objectives*. To develop a scoring system for evaluating the extrasynovial soft tissue inflammation of the shoulders in patients with polymyalgia rheumatica (PMR) and elderly-onset rheumatoid arthritis with PMR-like onset (pm-EORA) using ultrasound.* Methods*. We analyzed stored power Doppler (PD) images obtained by the pretreatment examination of 15 PMR patients and 15 pm-EORA patients. A semiquantitative scoring system for evaluating the severity of PD signals adjacent to the anterior aspect of the subscapularis tendon was designed.* Results*. A four-point scale scoring for the hyperemia on the subscapularis tendon was proposed as follows in brief: 0 = absent or minimal flow, 1 = single vessel dots or short linear-shape signals, 2 = long linear-shape signals or short zone-shape signals, or 3 = long zone-shape signals. This scoring system showed good intra- and interobserver reliability and good correlation to quantitative pixel-counting evaluation. By using it, we demonstrated that inflammation in PMR is dominantly localized in extrasynovial soft tissue as compared with pm-EORA.* Conclusions*. We proposed a reliable semiquantitative scoring system using ultrasound for the evaluation of extrasynovial soft tissue inflammation of the shoulders in patients with both PMR and pm-EORA. This system is simple to use and can be utilized in future investigations.

## 1. Introduction

Polymyalgia rheumatica (PMR), which presents with strong pain and stiffness around the shoulder and hip girdles, is a relatively common disease among adults aged ≥50 years [1]. Patients with PMR exhibit a very high inflammatory response and show dramatic improvement following the administration of corticosteroids [2]. Because there is no disease-specific marker, PMR is diagnosed based on its clinical manifestation and course [3]. Elderly-onset RA (EORA) patients often present polymyalgic symptoms that mimic PMR at the onset [4]. In cases in which serological markers are negative and arthritis in the peripheral small joints is lacking, discriminating EORA from PMR is sometimes very difficult, although treatment with antirheumatic drugs is necessary for the effective treatment of EORA.

In 2012, provisional classification criteria for PMR were published [5]. Unfortunately, these criteria were evaluated as useful in general but weak in discriminating between PMR and RA [5]. Two validation studies failed to conclude whether or not the optional use of musculoskeletal ultrasound (US) items in the new criteria improves the differential diagnosis between PMR and EORA [5, 6]. Both studies reported that the optional use of US items did not, however, improve the ability of the criteria to discriminate PMR from EORA with PMR-like onset (pm-EORA). In essence, binary assessment for the presence or absence of shoulder synovitis (tenosynovitis, bursitis, and joint synovitis) and hip synovitis (bursitis and joint synovitis) by US does not provide helpful information for distinguishing between PMR and pm-EORA.

What is the hallmark of the pathology detected by imaging modalities in shoulders with PMR? Studies using fat-saturated, contrast-enhanced magnetic resonance imaging (MRI) revealed soft tissue inflammation around the shoulder in addition to inflammation in the synovial tissues around the shoulder, such as tenosynovitis of the long head of biceps (LHB), subdeltoid/subacromial bursitis (SDB/SAB), and glenohumeral joint synovitis (GHJ), although no such inflammation in the extrasynovial soft tissues has been mentioned in previous studies employing US [7, 8].

Through clinical experience of the US examination of the shoulder lesions in untreated patients with PMR and pm-EORA, we noted that strong power Doppler (PD) signals indicating hyperemia are detected adjacent to the anterior aspect of the subscapularis tendon (SScT). We assume that this hyperemia on the SScT represents the extrasynovial soft tissue inflammation related to the polymyalgic feature. To clarify the clinical significance of this hyperemia, it is necessary to establish the method for evaluating it. In this study, we proposed and validated a semiquantitative scoring system for evaluating PD signals on the SScT in the shoulders of patients with PMR and pm-EORA. We also used a semiquantitative US scoring system for the three different components of synovitis in the shoulder, namely, tenosynovitis, bursitis, and joint synovitis, for the comprehensive assessment of the shoulder synovitis, in order to compare the severity of inflammation in extrasynovial soft tissue with that in synovial tissues.

## 2. Methods

### 2.1. Patients

This study was conducted in accordance with the principles of the Declaration of Helsinki. The medical records of patients who visited the hospital after January 2010 for examination of PMR-like symptoms were retrospectively reviewed to identify patients who fulfilled the following inclusion criteria: (i) musculoskeletal US was performed to evaluate persistent inflammatory pain and stiffness in the neck and shoulder girdle, regardless of pelvic girdle involvement; (ii) musculoskeletal US examination was performed before starting treatment with corticosteroids or antirheumatic drugs; (iii) follow-up clinical information one year after the US examination was available. By reviewing the clinical data during the one-year follow-up, including data on the resistance to corticosteroids alone, the need for and effectiveness of antirheumatic drugs, and the development of proliferative and/or bone-erosive synovitis in the peripheral small joints, the final diagnoses were confirmed by agreement among the attending physician and two rheumatologists (A. O. and Y. S.) certified by the Japan College of Rheumatology. Among patients who were examined between January 2010 and August 2013, 15 consecutive patients who were eventually diagnosed with PMR and 15 consecutive patients who were eventually diagnosed with pm-EORA were enrolled in this study for comparison. The 2012 EULAR/ACR provisional classification criteria for PMR and the 2010 ACR/EULAR classification criteria for RA were also tested to the patients [5, 9].

### 2.2. Clinical and Serological Data

At the time of US examination, clinical and serological data, including sex, age, disease duration, rheumatoid factor (RF) titer, anti-citrullinated peptide antibody (ACPA), C-reactive protein (CRP), and erythrocyte sedimentation rate (ESR), were available for all patients. Serum matrix metalloproteinase 3 (MMP-3) was available for 29 patients. CRP, RF, and MMP-3 were measured simultaneously within 10 days prior to US examination. RF was quantified by immunoturbidimetric assay (normal <15 U/ml; N-assay TIA RF; Nittobo Medical, Tokyo, Japan); ACPA was quantified by anti-CCP2 enzyme-linked immunosorbent assay (normal <4.5 U/ml; MESACUP CCP TEST, MBL; Nagoya, Japan); and MMP-3 was quantified by latex turbidimetric immunoassay (normal range, male: 36.9–121 ng/mL, female: 17.3–59.7 ng/mL; Panaclear MMP-3 Late; Daiichi Fine Chemicals, Takaoka, Japan).

### 2.3. US Image Acquisition for the Evaluation of Hyperemia on the SScT

**Figure 1:**
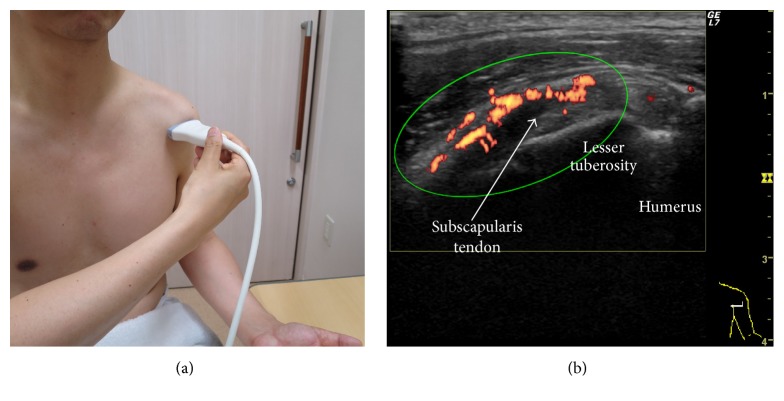
Scanning method for the evaluation of hyperemia adjacent to the anterior aspect of the subscapularis tendon. (a) Arm and probe position for the evaluation of the subscapularis tendon in the long axis. The shoulder is in a neutral position, the elbow is fixed to 90°, and the hand is spinated. The probe is placed perpendicular to the shoulder. (b) Long axis ultrasound view of the subscapularis tendon (arrow). The subscapularis tendon is superficial to the lesser tuberosity and medial to the bicipital groove. Note that the hypoechoic appearance of the medial part of the subscapularis tendon is due to the anisotropy. The area indicated by an ellipse, including the anterior soft tissues and the posterior tendon tissues and excluding the bicipital groove, is assessed for the severity of hyperemia.

US examinations were performed using a GE LOGIQ 7 device (GE Medical Systems; Milwaukee, WI) by an experienced examiner (T. S.). A 10 to 14 MHz linear transducer was used at 12.0 MHz for gray scale and 6.7 MHz for color mode. The hyperemia adjacent to the anterior aspect of the SScT was scanned in the horizontal long axis view with a neutral position (Figure 1(a)), because external rotation may lead to a decrease in blood flow possibly due to tension in the soft tissues. PD settings were identical to the preset parameters (pulse repetition frequency 1.0 kHz, Doppler gain 25) for every patient. Images with the most pronounced PD activity were identified from the cine-loop and stored.

### 2.4. Development and Validation of a Semiquantitative Scoring System

Stored images were used for the semiquantitative and quantitative evaluation. With a focus on the anterior aspect of the SScT, the area (including the anterior soft tissues and the posterior tendon tissues and excluding the intertubercular groove) was assessed for the severity of hyperemia (Figure 1(b), elliptic region). Based on a subjective evaluation, we developed a semiquantitative four-point scale scoring system.

In order to evaluate intra- and interobserver reliability, 40 selected images were randomized and rescored by the sonographer (T. S.) after a one-year interval and by another experienced sonographer/rheumatologist (Y. S.). Unweighted kappa statistics were calculated.

For validating the semiquantitative scoring, the same set of 40 images was used for quantitative evaluation. The images were opened in Adobe Photoshop elements 13, orange color pixels corresponding to the PD signals in the appropriate area were selected using the Magic Wand tool, and the pixel number was counted using the histogram panel. The area of signals was calculated in square millimeters with reference to the scale in the image. The relationship between quantitative measurement and semiquantitative scoring for hyperemia was plotted.

### 2.5. US Image Acquisition for the Evaluation of Synovial Pathologies

Shoulders were scanned according to a standardized scanning method [10]. With the shoulder in a neutral position, the glenohumeral joint (GHJ) was evaluated by transverse scanning of the posterior recess. This was complemented by dynamic observation during internal and external shoulder rotation. Similarly, with the shoulder in a neutral position, the long head of biceps (LHB) tendon sheath was evaluated by transverse and longitudinal scanning in the bicipital groove. The subdeltoid bursa (SDB) and subcoracoid bursa (SCB) were scanned with the shoulder in a neutral position, whereas the subacromial bursa (SAB) was scanned with the shoulder in a modified Crass position.

### 2.6. Grading and Scoring of Synovial Pathologies

All gray-scale US (GSUS) and power Doppler US (PDUS) findings for each synovial pathology were semiquantitatively graded and scored from 0 to 3 (0 = absent, 1 = mild, 2 = moderate, and 3 = severe) by analyzing the stored images, with the exception that a score of 0 could be given based on the description in a written report. All grading was done by the examiner (T. S.) who performed the US examinations. The GSUS and PDUS grading of GHJ synovitis was based on the SOLAR scoring system [11]. The GSUS and PDUS grading of LHB tenosynovitis was based on the OMERACT definition [12]. The GSUS grading of shoulder bursitis was subjectively determined (0 = absent, 1 = mild, 2 = moderate, and 3 = severe), whereas the PD signal of bursitis was subjectively graded on a semiquantitative scale (0 = absent or minimal flow, 1 = mild or single-vessel signal, 2 = moderate or confluent vessels, and 3 = severe or vessel signals in >50% of the synovium area). Although the GSUS and PDUS scores were determined for each instance of bursitis of the SDB, SAB, and SCB, those for shoulder bursitis were represented by the largest score among the three lesions. The shoulder synovitis score (SSS) was calculated as the sum of the GSUS and PDUS scores for the three pathologies (total of six scores) in each shoulder. The patient SSS (PSSS) was calculated as the sum of the scores for both shoulders of each patient.

### 2.7. Statistical Analysis

All statistical analyses were performed with EZR (Saitama Medical Center, Jichi Medical University, Saitama, Japan), which is a graphical user interface for R (The R Foundation for Statistical Computing, Vienna, Austria) [13]. The differences between the two groups were examined using Mann–Whitney *U* test. A correlation between two variables was examined using Spearman's rank correlation test. Statistical significance was set at a *p* value of less than 0.05. Intra- and interobserver reliability of the semiquantitative score were estimated using calculations of unweighted kappa statistics.

## 3. Results

### 3.1. Patient Demographics

Demographic and clinical data at the time of US examination are shown in Table 1. There were no significant differences in age and sex between the groups. Disease duration was shorter in the PMR group than in the pm-EORA group. Stiffness in the shoulder girdle was present in all patients in both groups. Pain in the bilateral shoulder was present in all patients in the PMR group and in the majority of patients in the pm-EORA group. Peripheral synovitis distal to the shoulder or knee was present in almost all patients in the pm-EORA group and in two patients in the PMR group. One patient in the PMR group tested positive for RF, whereas 60% of the patients in the pm-EORA group were seronegative. Both CRP and ESR were higher in the PMR group than in the pm-EORA group. Serum MMP-3 tended to be higher in the pm-EORA group than in the PMR group. Antirheumatic drugs including methotrexate were administered to all patients in the pm-EORA group, and methotrexate was administered to three patients in the PMR group as a corticosteroid-sparing agent during the one-year follow-up.

When the clinical data at the time of US examination were evaluated, all patients in the PMR group fulfilled the 2012 EULAR/ACR criteria for PMR. However, one-third of patients in the pm-EORA group did not fulfill the 2010 ACR/EULAR criteria for RA, possibly due to the low prevalence of seropositive patients. In addition, 60% of patients in the pm-EORA group had scores of 4 or higher for the 2012 PMR criteria, possibly because they presented with PMR-like onset. Therefore, it seems that the clinical data at the time of the US examination were not sufficient to predict the final diagnosis confirmed after one year of follow-up.

### 3.2. Development of a Semiquantitative Four-Point Scale Scoring System

**Figure 2:**
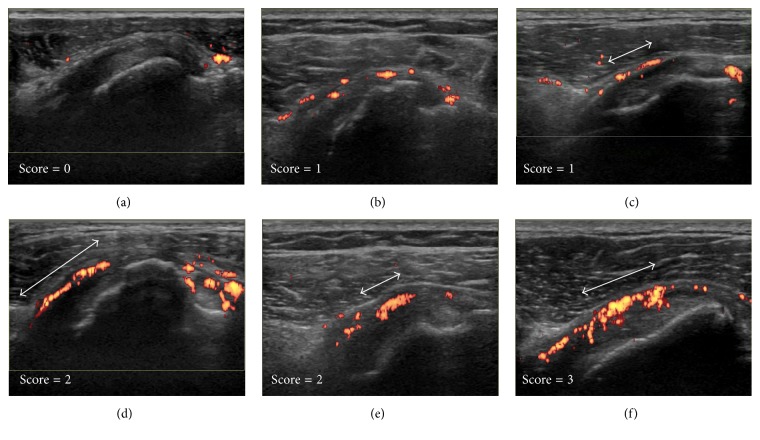
Illustrative ultrasound images for the four-point scale scoring of the hyperemia on the subscapularis tendon. Score 0 = absent or minimal flow (a), score 1 = single vessel dots (b), or confluent linear shape signals shorter than half the length of the region to be evaluated (c), score 2 = confluent linear shape signals longer than half the length of the region to be evaluated (d), or confluent zone shape signals shorter than half the length of the region to be evaluated (e), score 3 = confluent zone shape signals longer than half the length of the region to be evaluated (f). Double arrows indicate the length of the linear shape signals (c, d), or the length of the zone shape signals (e, f).

Based on a subjective evaluation, we proposed a semiquantitative four-point scale scoring system for the severity of the hyperemia adjacent to the anterior aspect of the SScT as follows. Illustrative images are shown in Figure 2. Score 0 = absent or minimal flow (Figure 2(a)); score 1 = single vessel dots (Figure 2(b)) or confluent linear shape signals shorter than half the length of the region to be evaluated (Figure 2(c)); score 2 = confluent linear shape signals longer than half the length of the region to be evaluated (Figure 2(d)) or confluent zone shape signals shorter than half the length of the region to be evaluated (Figure 2(e)); or score 3 = confluent zone shape signals longer than half the length of the region to be evaluated (Figure 2(f)). The score was denominated as the hyperemia on subscapularis tendon score (HSScTS) and the sum of the scores of bilateral shoulders was denominated as Bil-HSScTS.

### 3.3. Validation of HSScTS Scoring System

**Figure 3:**
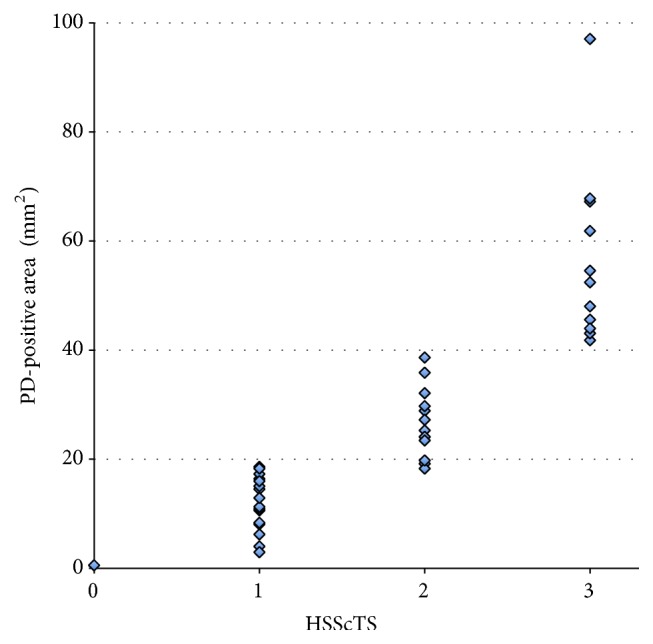
Correlations between the hyperemia on the subscapularis tendon score (HSScTS) and the area of power Doppler- (PD-) positive pixels among the 30 patients with polymyalgic symptoms.

The intraobserver unweighted kappa statistic obtained by rescoring 40 images blindly was 0.852. The interobserver unweighted kappa statistic was 0.745. The relationship between the quantitative measurement of PD-positive pixel areas and the semiquantitative HSScTS was plotted in Figure 3. These data indicated the strong reliability of the semiquantitative four-point scale scoring system for HSScTS.

### 3.4. Differences in Hyperemia on Subscapularis Tendon between Diseases or Clinical Conditions

The distribution of the HSScTS in the shoulders among each disease group is shown in Table 2. As control groups, 26 shoulders of 15 consecutive new-onset RA patients without a polymyalgic feature and 22 shoulders of 19 consecutive non-RA patients without a polymyalgic feature in whom HSScTS were recorded for symptomatic shoulders by US before starting treatment with corticosteroids or antirheumatic drugs were chosen. The non-RA control group included 5 shoulders with glenohumeral osteoarthritis, 2 shoulders with synovitis, acne, pustulosis, hyperostosis, and osteitis (SAPHO) syndrome, 2 shoulders with a rotator cuff tear, and 12 others. A HSScTS of higher than 1 was equally common among the two groups presenting a polymyalgic feature although it was rare among the control groups.

**Figure 4:**
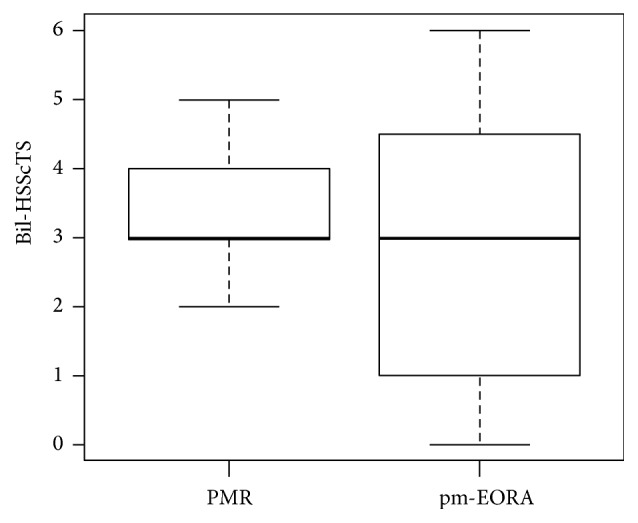
Comparison of the sum of the hyperemia on the subscapularis tendon scores of bilateral shoulders (Bil-HSScTS) between patients with PMR and pm-EORA.

Next, we compared the Bil-HSScTS, which is the sum of the HSScTS of bilateral shoulders. Bil-HSScTS showed no significant differences between the PMR group (median, 3; min–max [2–5]) and the pm-EORA group (median, 3; min–max [0–6]) (Figure 4). These results suggest that the hyperemia on the SScT is specific not to PMR but to the polymyalgic conditions.

### 3.5. Correlation between Severity of the Hyperemia on the SScT and Serum Markers for Inflammation

**Figure 5:**
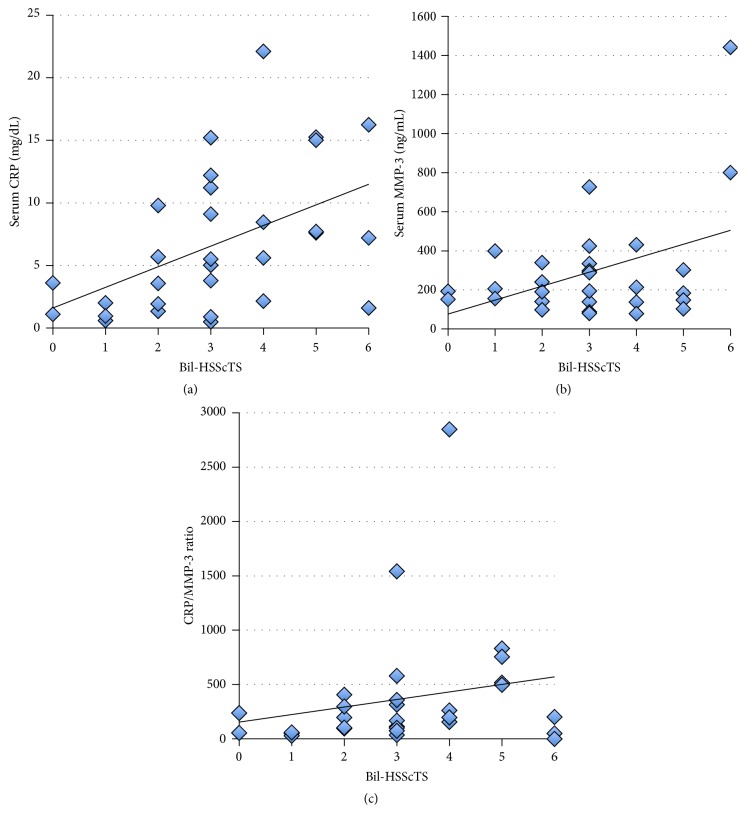
Correlations between the sum of the hyperemia on the subscapularis tendon scores of bilateral shoulders (Bil-HSScTS) and serum markers for inflammation. (a) Correlation between Bil-HSScTS and serum CRP. (b) Correlation between Bil-HSScTS and serum matrix metalloproteinase 3 (MMP-3). (c) Correlation between Bil-HSScTS and CRP/MMP-3 ratio. CRP/MMP-3 ratio was calculated by dividing serum CRP concentration by serum MMP-3 concentration.

Correlations between Bil-HSScTS and serum markers for inflammation were assessed among 30 patients from both the PMR and pm-EORA groups. As shown in Figure 5(a), Bil-HSScTS positively correlated with serum CRP (*R* = 0.522, *p* = 0.00309). In contrast, as shown in Figure 5(b), Bil-HSScTS did not correlate with serum MMP-3 (*R* = 0.121, *p* = 0.531). Assuming that the levels of serum CRP are related to the total sum of synovial inflammation and extrasynovial inflammation and that the levels of serum MMP-3 are related to synovial inflammation, we set up a new index, the CRP/MMP-3 ratio, which was defined as serum CRP concentration divided by serum MMP-3 concentration. As shown in Figure 5(c), Bil-HSScTS positively correlated with the CRP/MMP-3 ratio in the patients (*R* = 0.425, *p* = 0.0215). It is intriguing to speculate that Bil-HSScTS is related to extrasynovial inflammation and that the CRP/MMP-3 ratio can be used as a marker for the extent of extrasynovial inflammation compared to the extent of synovial inflammation.

### 3.6. Grading and Scoring of Each Synovial Pathology

**Figure 6:**
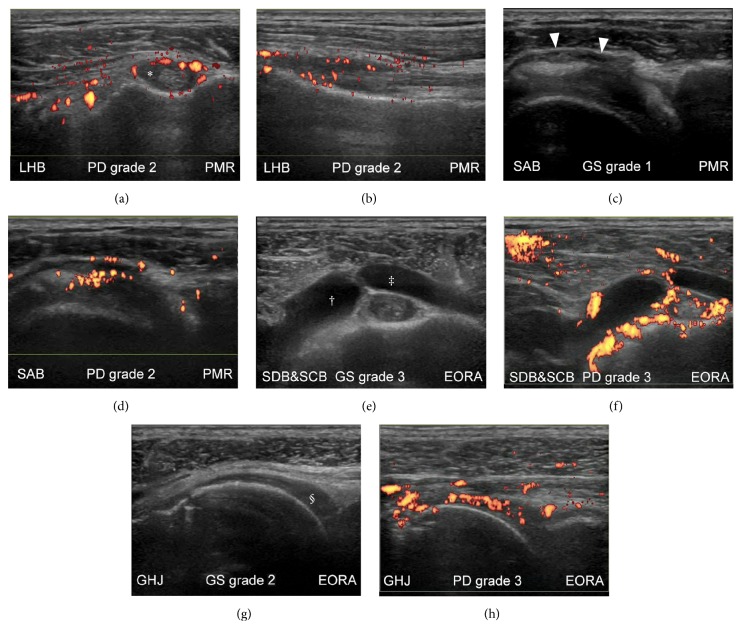
Representative ultrasound (US) images for grading of synovial pathology of the shoulder. Images from the shoulders of patients with polymyalgia rheumatica (PMR) (a–d) and elderly-onset rheumatoid arthritis (EORA) with PMR-like onset (e–h) are shown on gray-scale (c, e, and g) and power Doppler (a, b, d, f, and h) ultrasonograms. The shoulder was in a neutral position (a, b, e, f, and h), in a modified Crass position (c, d), or internally rotated (g). Moderate hyperemia was detected in the long head of biceps tendon sheath (*∗*) on transverse (a) and longitudinal (b) scans. Mild synovial effusion (c) and moderate hyperemia (d) were detected in the subacromial bursa (arrowheads). Severe synovial effusion (e) and severe hyperemia (f) were detected in the subcoracoid bursa (†) and subdeltoid bursa (‡). Moderate synovial proliferation (g) and severe hyperemia (h) were detected in the glenohumeral joint (§).

For the quality control of our US scoring of shoulder synovitis, we evaluated the kappa value of intra- and interobserver reliability by blindly rescoring 184 images. The intraobserver agreement was excellent with an unweighted kappa statistic of 0.844. The interobserver agreement was good with an unweighted kappa statistic of 0.675. While reports on the concordance rate of semiquantitative grading for shoulder bursitis are scarce, intra- and interobserver agreement were higher for shoulder bursitis, as compared with LHB tenosynovitis or GHJ synovitis (Table 3). Representative US images for the grading of the synovial pathology are shown in Figure 6.

The distribution of the GSUS and PDUS grades for the three kinds of synovial pathologies is shown in Table 4. Analysis of the distribution of the grades by Fisher's exact test revealed that the GS- and PD-grades of bursitis in the pm-EORA group were significantly higher than those in the PMR group. Comparison of the scores by Mann–Whitney *U* test showed similar findings. The GS-scores of bursitis in the pm-EORA group (median, 1; min–max [0–3]) were significantly higher than those in the PMR group (median, 0; min–max [0–3]; *p* = 0.0167). The PD-scores of bursitis in the pm-EORA group (median, 1; min–max [0–3]) were significantly higher than those in the PMR group (median, 0; min–max [0–2]; *p* = 0.0103). Whereas shoulder bursitis is generally thought to be a hallmark of PMR, our data obtained by semiquantitative comparison between PMR and pm-EORA revealed that bursitis was less severe in PMR than in pm-EORA.

### 3.7. Comprehensive Scoring of Synovial Inflammation for Each Shoulder or Patient

**Figure 7:**
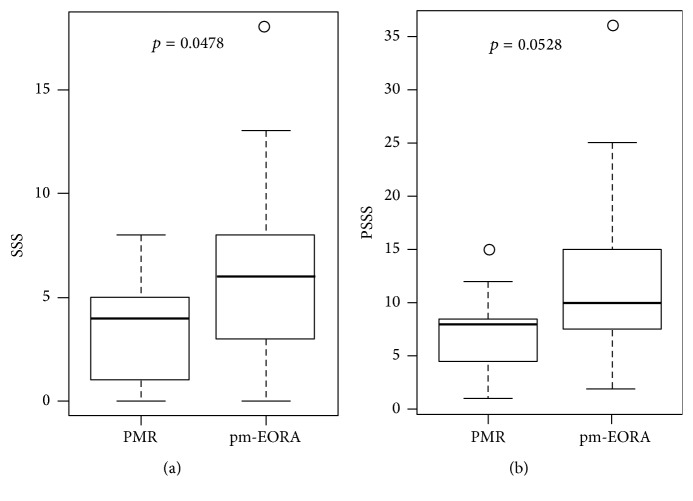
Comprehensive score for synovial inflammation for each shoulder or patient. The shoulder synovitis scores (SSS) (a) and patient shoulder synovitis scores (PSSS) (b) were compared between patients with PMR and those with EORA with PMR-like onset (pm-EORA) by Mann–Whitney *U* test.

The SSS was calculated as the sum of the GSUS and PDUS scores for the three pathologies (total of six scores) in each shoulder, and the PSSS was calculated as the sum of the SSS for both shoulders of each patient. As shown in Figure 7, comparison by Mann–Whitney *U* test revealed that the SSS was significantly higher in the pm-EORA group (median, 6; min–max [0–18]) than in the PMR group (median, 4; min–max [0–8]; *p* = 0.0478). However, the difference in the PSSS between the two groups did not reach the significance level (pm-EORA group median, 10; min–max [2–36] vs PMR group median, 8; min–max [1–15]; *p* = 0.0528). These results suggested that shoulder synovitis in PMR tends to be mild as compared with that observed in pm-EORA, possibly because synovitis in PMR may be characterized as exudative synovitis rather than proliferative synovitis as compared with synovitis in RA.

### 3.8. The Ratio of Bil-HSScTS to PSSS

**Figure 8:**
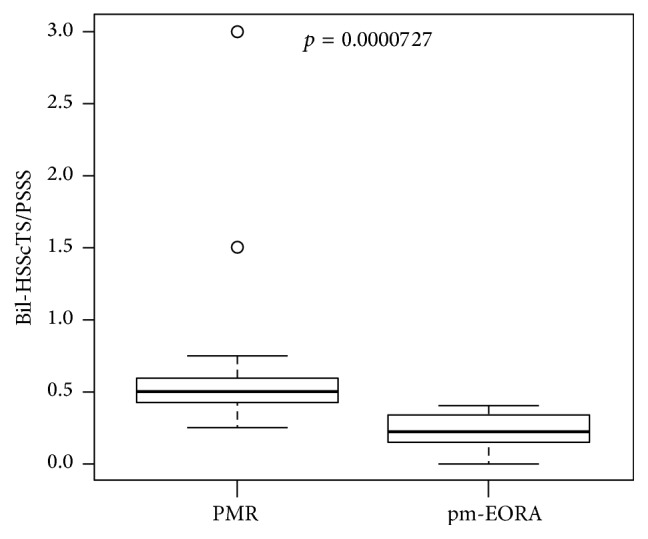
The ratio of the sum of the hyperemia on the subscapularis tendon scores of bilateral shoulders (Bil-HSScTS) to patient shoulder synovitis scores (PSSS). The ratio of Bil-HSScTS to PSSS was compared between patients with PMR and those with EORA with PMR-like onset (pm-EORA) by Mann–Whitney *U* test.

We set up another new index, the Bil-HSScTS/PSSS ratio, which was defined as Bil-HSScTS divided by PSSS. As shown in Figure 8, Bil-HSScTS/PSSS ratio was significantly much higher in the PMR group (median, 0.500; min–max [0.250–3.00]) than in the pm-EORA group (median, 0.214; min–max [0–0.400]; *p* = 0.0000727). Because we assume that the Bil-HSScTS/PSSS ratio represents the ratio of the severity of the inflammation in extrasynovial soft tissues to the severity of the inflammation in synovial tissues, it is suggested that the inflammation in PMR is dominantly localized in extrasynovial soft tissues as compared with that in pm-EORA.

### 3.9. Changes in the Hyperemia on the SScT after Treatment with Corticosteroids and/or Antirheumatic Drugs

**Figure 9:**
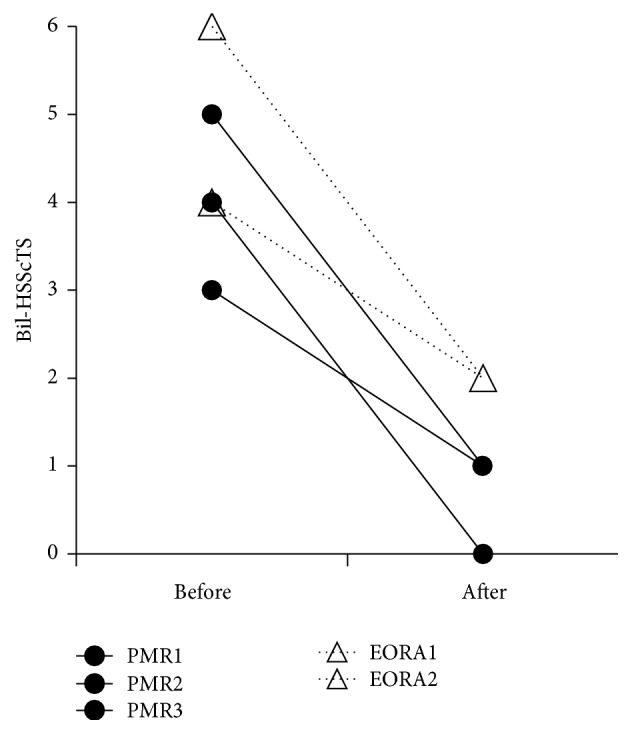
Changes in the sum of the hyperemia on subscapularis tendon scores of bilateral shoulders (Bil-HSScTS) after treatment with corticosteroids and/or antirheumatic drugs. Bil-HSScTS decreased after treatment in both PMR patients (circle, solid line) and EORA patients with PMR-like onset (triangle, dashed line).

Follow-up data on Bil-HSScTS after starting treatment with corticosteroids and/or antirheumatic drugs were available in only 5 of 30 patients (3 with PMR and 2 with pm-EORA). The intervals between the examinations varied considerably from 0.9 to 8.7 months. In all five cases, Bil-HSScTS decreased after treatment (Figure 9).

## 4. Discussion

Two groups have employed traditional MRI, fat-saturated MRI, and contrast-enhanced MRI in studying the primary site of inflammation in PMR since around 2000. Cantini et al. advocated that the primary site in PMR was the extracapsular synovial tissue, that is, the synovial bursa [14–16]. In contrast, McGonagle et al. argued that the primary site of inflammation was detected in nonsynovial soft tissues around the joint capsules and proposed that capsulitis/enthesitis, including inflammation of the functional enthesis, was the primary pathology in PMR and that synovitis occurred secondarily [17–20]. Currently, it remains unknown whether synovitis in the joints and their surroundings (tenosynovitis/bursitis) or capsule-/enthesis-based pathologies are the primary and secondary sites of PMR. Nevertheless, inflammation in PMR appears to be present in nonsynovial soft tissues, and this additional inflammatory element to synovitis likely contributes to the polymyalgic symptoms in PMR. Recent studies from Japan that employed fat-saturated MRI also confirmed that inflammation of both the synovium and extrasynovial soft tissues around the joints were present in PMR [7, 8].

In this study, we indicated that significant hyperemia was often detected adjacent to the anterior aspect of the SScT in the shoulders with PMR. It is very likely that this hyperemia detected by PDUS corresponds to the extrasynovial soft tissue inflammation depicted by MRI. Moreover, this region on the SScT may represent the functional enthesis that is possibly affected in PMR. Because the moderate to severe hyperemia was equally detected in both PMR and pm-EORA, the hyperemia on the SScT is specific not to PMR but to the polymyalgic features common to both diseases. Presumably, other interfaces between the muscle and the rotator cuff, such as the supraspinatus tendon or the infraspinatus tendon, can be candidate sites for detecting extrasynovial inflammation. However, the anterior aspect of the subscapularis tendon is probably the optimal site because there are “echo windows” that allow good penetration by US.

We proposed the first tool utilizing US for evaluating soft tissue inflammation, which is the key feature of the polymyalgic symptoms around the shoulder. Our semiquantitative four-point scale scoring system for the severity of the hyperemia on the SScT is not only simple and easy to use, but also well standardized. The system showed good intra- and interobserver reliability and good correlation to quantitative pixel-counting evaluation. Using this scoring system along with the comprehensive scoring system for shoulder synovitis, we demonstrated that Bil-HSScTS/PSSS ratio was significantly higher in the PMR group than in the pm-EORA group. It suggests that the inflammation in PMR is dominantly localized in extrasynovial soft tissue as compared with that in pm-EORA.

This scoring system can be utilized for many investigations, including (i) correlation between the hyperemia on the SScT and stiffness or range of motion in the shoulders with PMR and (ii) association between the extent of the hyperemia on the SScT and the necessity for corticosteroid therapy. Unfortunately, we could only show very limited data for the change in hyperemia on the SScT before and after treatment. Longitudinal studies revealing the characteristics and the significance of the changes in the hyperemia on the SScT are needed.

## 5. Conclusions

In this study, we proposed the first tool utilizing US for the evaluation of extrasynovial soft tissue inflammation in the shoulders of patients with both PMR and pm-EORA. This semiquantitative four-point scale scoring system with high reliability is simple to use and can be utilized in future investigations.

## Figures and Tables

**Table 1:** Demographic and clinical characteristics of patients at the time of US examination.

Diagnosis^a^	PMR	pm-EORA
Number of patients	15	15
Age (years)^b^	72.6 ± 7.7	70.7 ± 7.0
Sex (female)	33.3%	46.7%
Disease duration (months)^b^	1.7 ± 0.8	2.7 ± 1.1
Shoulder girdle stiffness	100%	100%
Bilateral shoulder pain	100%	86.7%
Peripheral joint involvement	13.3%	93.3%
Positive RF	6.70%	33.3%
Positive ACPA	0.0%	33.3%
Positive RF and/or ACPA	6.7%	40.0%
CRP (mg/dL)^b^	9.3 ± 5.3	4.2 ± 5.0
ESR (mm/h)^b^	102 ± 27	79 ± 30
MMP-3 (ng/ml)^b^	255 ± 174	333 ± 366^c^
2012 EULAR/ACR PMR criteria score ≧ 4	100%	60%
2010 ACR/EULAR RA criteria score ≧ 6	0.0%	66.6%
Initiation of antirheumatic drugs during one-year follow-up^a^	20%	100%

a, data after one-year follow-up; b, mean ± standard deviation; c, *n* = 14; ACPA, anti-citrullinated peptide antibody; CRP, C-reactive protein; ESR, erythrocyte sedimentation rate; MMP-3, matrix metalloproteinase 3; pm-EORA, elderly-onset rheumatoid arthritis with PMR-like onset; PMR, polymyalgia rheumatica; RF, rheumatoid factor; US, ultrasound.

**Table 2:** Distribution of the hyperemia on subscapularis tendon scores (HSScTS) in the shoulders among each disease group.

	Score 0	Score 1	Score 2	Score 3
PMR (*n* = 30)	6.7%	36.7%	36.7%	20.0%
pm-EORA (*n* = 30)	26.7%	26.7%	23.3%	23.3%
RA control (*n* = 26)	57.7%	38.5%	3.8%	0.0%
Non-RA control (*n* = 22)	40.9%	59.0%	0.0%	0.0%

PMR, polymyalgia rheumatica; pm-EORA, elderly-onset rheumatoid arthritis with PMR-like onset.

**Table 3:** Intra- and interobserver reliability evaluated by rescoring 184 images blindly shown as unweighted kappa statistics.

Pathology and mode	Number of the images evaluated	Intraobserver	Interobserver
LHB (GS & PD)	72	0.78	0.626
Bursa (GS & PD)	72	0.926	0.72
GHJ (GS & PD)	40	0.753	0.616
GS (all pathologies)	92	0.807	0.657
PD (all pathologies)	92	0.886	0.69
Overall	184	0.844	0.675

LHB, long head of biceps tendon sheath; GHJ, glenohumeral joint; GS, gray scale; PD, power Doppler.

**Table 4:** Distribution of GS and PD grades for three kinds of shoulder synovial pathologies.

	PMR				pm-EORA				Fisher's exact test
	*n* = 30				*n* = 30				
Grade (score)	0	1	2	3	0	1	2	3	*p* value
LHB GS (%)	20.0	46.7	30.0	3.3	30.0	26.7	26.7	16.7	0.176
LHB PD (%)	33.3	26.7	36.7	3.3	33.3	13.3	30.0	23.3	0.108
Bursa GS (%)	60.0	30.0	6.7	3.3	40.0	13.3	23.3	23.3	0.0157
Bursa PD (%)	76.7	10.0	13.3	0.0	46.7	16.7	20.0	16.7	0.0424
GHJ GS (%)	80.0	16.7	3.3	0.0	66.7	10.0	16.7	6.7	0.141
GHJ PD (%)	86.7	10.0	3.3	0.0	70.0	20.0	0.0	10.0	0.104

GHJ, glenohumeral joint; GS, gray scale; LHB, long head of biceps tendon sheath; PD, power Doppler; pm-EORA, elderly-onset rheumatoid arthritis with PMR-like onset; PMR, polymyalgia rheumatic.
